# Cerebral Developmental Abnormalities in a Mouse with Systemic Pyruvate Dehydrogenase Deficiency

**DOI:** 10.1371/journal.pone.0067473

**Published:** 2013-06-26

**Authors:** Lioudmila Pliss, Kathryn A. Hausknecht, Michal K. Stachowiak, Cynthia A. Dlugos, Jerry B. Richards, Mulchand S. Patel

**Affiliations:** 1 Department of Biochemistry, School of Medicine and Biomedical Sciences, University at Buffalo, The State University of New York, Buffalo, New York, United States of America; 2 Research Institute on Addictions, University at Buffalo, The State University of New York, Buffalo, New York, United States of America; 3 Department of Pathology and Anatomical Sciences, School of Medicine and Biomedical Sciences, University at Buffalo, The State University of New York, Buffalo, New York, United States of America; Hôpital Robert Debré, France

## Abstract

Pyruvate dehydrogenase (PDH) complex (PDC) deficiency is an inborn error of pyruvate metabolism causing a variety of neurologic manifestations. Systematic analyses of development of affected brain structures and the cellular processes responsible for their impairment have not been performed due to the lack of an animal model for PDC deficiency. METHODS: In the present study we investigated a murine model of systemic PDC deficiency by interrupting the X-linked *Pdha1* gene encoding the α subunit of PDH to study its role on brain development and behavioral studies. RESULTS: Male embryos died prenatally but heterozygous females were born. PDC activity was reduced in the brain and other tissues in female progeny compared to age-matched control females. Immunohistochemical analysis of several brain regions showed that approximately 40% of cells were PDH^−^. The oxidation of glucose to CO_2_ and incorporation of glucose-carbon into fatty acids were reduced in brain slices from 15 day-old PDC-deficient females. Histological analyses showed alterations in several structures in white and gray matters in 35 day-old PDC-deficient females. Reduction in total cell number and reduced dendritic arbors in Purkinje neurons were observed in PDC-deficient females. Furthermore, cell proliferation, migration and differentiation into neurons by newly generated cells were reduced in the affected females during pre- and postnatal periods. PDC-deficient mice had normal locomotor activity in a novel environment but displayed decreased startle responses to loud noises and there was evidence of abnormal pre-pulse inhibition of the startle reflex. CONCLUSIONS: The results show that a reduction in glucose metabolism resulting in deficit in energy production and fatty acid biosynthesis impairs cellular differentiation and brain development in PDC-deficient mice.

## Introduction

The pyruvate dehydrogenase complex (PDC) plays a pivotal role in glucose metabolism by converting pyruvate to acetyl-CoA and linking the glycolytic pathway with the tricarboxylic acid cycle. Mammalian PDC, a multienzyme mitochondrial complex, is composed of multiple copies of three catalytic components [pyruvate dehydrogenase (PDH; α_2_β_2_ tetramer), dihydrolipoamide acetyltransferase and dihydrolipoamide dehydrogenase], a non-catalytic component dihydrolipoamide dehydrogenase-binding protein and two regulatory components [pyruvate dehydrogenase kinases (four isoenzymes) and phosphopyruvate dehydrogenase phosphatases (two isoenzymes)] [1], [2]. Regulation of PDC is exerted by its reversible phosphorylation (inactivation) and dephosphorylation (activation) by its specific kinases and phosphates, respectively [1], [2]. All of the catalytic and regulatory subunits are encoded by single copies of autosomal genes with the exception of the α subunit of PDH. In mammals, PDHα is encoded by two genes: an X-linked allele (*PDHA1* in human and *Pdha1* in mouse) that is expressed in somatic cells and an autosomal, intronless paralogue (*PDHA2* in human; *Pdha2* in mouse) that is expressed only in post-meiotic spermatogenic cells [3], [4]. To allow a high rate of aerobic glucose oxidation, adult mammalian brain maintains a high proportion (∼70%) of PDC in the dephosphorylated (active) form [5]. During the prenatal and early postnatal periods PDC also plays a central role in lipid biosynthesis from glucose in the brain [6].

PDC deficiency is one of major genetic disorders of oxidative metabolism, resulting in congenital lactic acidosis and extremely heterogeneous clinical manifestations, which are limited largely to the central nervous system [7]–[10]. The large majority of all reported PDC deficiency cases involve defects in the subunit of the PDH component of the complex [8]–[14]. More than 371 cases of PDC deficiency have been reported and more than 80% of these cases involve defects in PDH [10]. Various congenital cerebral malformations and neurological dysfunctions have been reported in affected patients. These may range from mild ataxia to profound psychomotor retardation and even early postnatal death. Gross congenital brain malformations have been described in PDC deficiency cases, such as microcephaly, cerebral atrophy and abnormal development of the corpus callosum and pyramids [7], [15]–[18]. Because of the location of *PDHA1 on the* X chromosome, affected males and females manifest the disease differently. Male patients usually develop severe systemic lactic acidosis and neural defects that lead to lethality during early childhood. From the few reported cases of heterozygous females who carried mutations in one of their *PDHA1* alleles, it appears that the disease generally tends to be less severe [7], [16], [19]. The severity of symptomatology in females can vary tremendously, even when individuals carry the same mutation. The inter-individual variability has been attributed to differences in patterns of X chromosome inactivation between individuals [19], [20].

Despite identification of a large number of mutations in the *PDHA1* gene, impairment at the cellular level in affected PDC-deficient patients remains largely uncharacterized. Previous studies investigating this metabolic disorder have been limited due to the lack of availability of a suitable animal model [21]. We have developed a murine model that carries a mutation in the X-linked *Pdha1* gene, the orthologue of the human *PDHA1* gene [22]. Earlier we developed a murine model of PDC deficiency that expressed PDC deficiency in the brain only (termed as ‘cerebral’ PDC deficiency) [23]. These PDC-deficient female mice developed brain structural abnormalities somewhat similar to those observed in female patients with this disorder, including underdevelopment of white matter structures and gross reduction of white matter. The ‘systemic’ PDC deficiency model reported here also develops similar brain structural abnormalities to that observed in the ‘cerebral’ PDC-deficient murine model [23], and more importantly it resembles closely to PDC deficiency observed in female patients developing PDC deficiency during early embryogenesis and expressing in all tissues. Additionally, in the present study we have addressed the question of the developmental origin of brain defects characteristic for systemic PDC deficiency by monitoring proliferation, migration and differentiation of neuronal cells during development.

## Materials and Methods

### Generation of PDC-deficient Mice

The University at Buffalo’s Animal Care Program and Laboratory Animal Facilities are fully accredited by the Association for Assessment and Accreditation of Laboratory Animal Care (AAALAC) International. The Institutional Animal Care and Use Committee at the University at Buffalo approved all animal procedures (protocol # BCH11064N). Generation of a mouse colony harboring a silent mutation in the *Pdha1* gene (two loxP sites into intron sequences flanking exon 8; referred to as the *Pdha1^flox8^* allele) was reported earlier [22]. These mice were maintained on a standard rodent laboratory diet and water *ad libitum*. To initiate deletion of exon 8 *in vivo* in all tissues of the progeny, homozygous floxed females (genotypes: *Pdha1^flox8^/Pdha1^flox8^*) were bred with homozygous males from an *EIIa-Cre* transgenic mouse line (genotype: *Pdha1^wt^/Y*; *Cre^all+^*; referred to as Cre transgenic males) [24] to generate experimental heterozygous female progeny (referred to as PDC-deficient females with the genotype: *Pdha1^wt^/PDHa1* ^Δ*ex8*^ *, Cre^all+^*). The transgenic *Cre^all+^* mouse strain was homozygous for an autosomally integrated *Cre* transgene under the control of the adenovirus *EIIa* promoter that targets expression of Cre recombinase beginning on embryonic day 1 [24]. To generate control female progeny (referred to as controls) wild-type males (without carrying a *Cre* transgene), purchased from the Jackson Laboratories (Bar Harbor, ME), were bred with homozygous *Pdha1^flox8^* females. Progeny were nursed by their natural mothers, weaned onto a standard rodent laboratory diet and water *ad libitum* on postnatal day 21. Mice were anesthetized (ketamine/xylazine, 80 mg and 10 mg/kg body weight, respectively) and killed at different ages for tissue harvesting. Samples of brain, liver and skeletal muscle were rapidly removed, processed according to the protocol described below. Processed samples were stored frozen until analyzed.

### Genotyping

The progeny were tested for the presence or absence of the three *Pdha1* alleles (*Pdha1^wt^, Pdha1^flox8^, and Pdha1* ^Δ*ex8*^) and the *Cre* transgene by PCR analyses using tail clip DNA samples close to postnatal day 15 (P15), and reconfirmed using DNA isolated from central nervous system tissue after tissue harvesting [23]. Genomic DNA was isolated using the Omni Prep kit (Bio-WORLD, Dublin, OH) and subjected to genotyping by PCR using specific primer sets and conditions as previously described [22], [23].

### PDC Activity Determination

PDC exists as ‘active’ (dephosphorylated form) and ‘inactive’ (phosphorylated form), and its inter-conversion between these two forms can readily alter the flux through this complex. Hence it is appropriate to measure both the activities to show that both the activity levels are decreased in PDC-deficient mice. ‘Active’ and ‘total’ PDC activities were measured as described previously in thrice freeze-thawed homogenates of brain, liver and skeletal muscle of mice on embryonic (E) day18 and/or postnatal (P) days 0.5, 15 and 35 [22], [23], [25]. PDC activity was determined by production of ^14^CO_2_ from [1-^14^C]pyruvate. For ‘active’ PDC activity, homogenizing buffer contained dichloroacetate (inhibitor of PDH kinases) and sodium fluoride (inhibitor of PDH phosphatases) to preserve the ‘active’ PDC activity. For measurement of ‘total’ PDC activity, purified recombinant PDH phosphatase 1 was added to freeze-thawed homogenates and incubated for 30 min to dephosphorylate phospho-PDH [22], [23]. PDC activity is expressed as munits/mg protein.

### Measurements of Substrate Oxidation and Lipogenesis

Oxidation to ^14^CO_2_ and incorporation of the carbon from radiolabeled glucose or acetate into fatty acids were assessed in tissue slices from brain at P15 and P35 as previously described [6], [22], [23]. Tissue slices were incubated in a sealed flask containing 3 ml of Krebs-Ringer bicarbonate buffer, pH 7.4 containing 10 mM [U-^14^C]glucose (Amersham Pharmacia Biotech, Piscataway, NJ) or 10 mM [1,2-^14^C]acetate (ICN Biomedicals, Inc., Aurora, OH) plus 10 mM unlabeled glucose for 2 h at 37°C. The trapping of ^14^CO_2_ and extraction of lipids were performed as described previously [6]. The results are expressed as nmol of radioactive substrate oxidized to ^14^CO_2_ or incorporated into fatty acids per gram of wet tissue weight per h.

### Measurements of Plasma Substrates

Blood samples from P35 PDC-deficient and control females were collected in EDTA coated tubes and plasma L-(+)-lactate was measured spectrophotometrically [26].

### Tissue Preparation and Structural Analyses

Brains of P35 female progeny were fixed by perfusion with 4% paraformaldehyde in 0.1 M phosphate buffered saline (PBS) (pH 7.2) as described previously [22], [23]. Brains were then postfixed overnight and subsequently processed in 10%, 20% and 30% sucrose in 0.1 M PBS and frozen in Cryo-M-Bed embedding compound (Bright Instrument Company, Huntingdon, UK**).** Five sets of coronal 20 µm sections were cut with a Vibratome and collected as consecutive pair on gelatin/poly-L-lysine coated glass slides. One set of sections was stained with 0.2% Cresyl violet in sodium acetate (pH 4.5) to reveal nuclei of neuronal and glial cells. Following dehydration and mounting with Permount, these slides were analyzed with a Zeiss Axiovert 35 microscope. The second set of brain sections was immunostained with anti-PDHα antibody (mouse monoclonal, MitoSciences, Eugene, OR). These sections were rinsed with PBS, blocked with normal goat serum for 30 min, and then incubated with the primary antibody (dilution 1∶100) overnight. After a wash with PBS, Alexa Fluor 488 secondary antibody (goat-anti-mouse Alexa Fluor 488, Molecular Probes, Carlsbad, CA) was applied for 2 h. After a rinse in PBS slides were mounted with Immu-Mount (Thermo Electron Corporation, Waltham, MA).

### Immunolabeling of Cellular Markers

Additional brain sections from P35 females were subjected to co-staining with 2 to 3 different antibodies in order to reveal the cell phenotype. The following markers were used: neuronal cell nuclear marker (NeuN, dilution 1: 500, mouse monoclonal, Chemicon, Temecula, CA), calretinin, specific for cerebellar granule cells (1∶1000, mouse monoclonal, Chemicon, Temecula, CA) and calbindin D-28K, specific for cerebellar Purkinje neurons (1∶2000, mouse monoclonal, Sigma-Aldrich Corporation, St. Louis, MO). Secondary antibodies were goat-anti-rat Alexa Fluor 594 (for BrdU) and goat-anti-mouse Alexa Fluor 488 (for PDHα, NeuN, calbindin) (Molecular Probes, Invitrogen, Carlsbad, CA).

### Quantification of Densities of PDHα^+^ and PDHα^−^ Cells

Ten sections from P35 control and PDC-deficient females (n = 2) were selected at systematic intervals as stated below and stained with anti-PDHα antibody [27]. Sections were spaced at 120 µm apart and the following brain regions were identified according to the atlas of Paxinos and Watson [28]. These regions included the dorsomedial neocortex (1.18 to −0.82 from bregma), the subventricular zone of III ventricle (0.38 to −2.18 from bregma), CA1 of the hippocampus (−1.22 to −2.30 from bregma), the striatum, (1.18 to −0.82 from bregma) and crus1, the ansiform and simple lobules of the cerebellum (−5.68 to −6.96 from bregma). Digital images of these brain areas were taken using an unbiased approach [29], [30] from either right or left hemisphere (or both for hypothalamus) with an AxioImager Zeiss (Zeiss Axiovert 35) microscope. Axioimaget LE Rel. 4.5 Program was used to calibrate images and a 2500 µm^2^ square counting grid (50 µm×50 µm) was applied over randomly selected regions of the dorsomedial neocortex, hypothalamus, hippocampus and striatum. For the cerebellum, a rectangular grid (100 µm×50 µm) was applied over a randomly selected region containing the Purkinje neuron layer. Only cells with a visible and in focus nucleus were counted. The density of PDHα^+^ and of PDHα^−^ cells was determined separately.

### BrdU Labeling Experiments in the Prenatal and Postnatal Periods

To monitor proliferation and differentiation of cells in the developing brain, two sets of BrdU-labeling experiments were performed at E14 and at P5. To label the pool of proliferating cells in fetal brain at the E14 stage and their differentiation into neurons by the postnatal day 5, bromodeoxyuridine (BrdU; Sigma-Aldrich Corporation, St. Louis, MO) in PBS was injected intraperitoneally into pregnant dams (50 mg/kg body weight; 3 times 2 h apart) on gestation day 14 [31]. Dams were allowed to deliver and nurse their progeny. P5 female progeny brains were processed as described above and sections were co-immunostained for BrdU and NeuN [32]. Briefly, brains were embedded in gelatin and lightly post-fixed overnight. The brains were completely sectioned at 30 µm in the coronal plane, saving all sections for stereological counting. To detect the BrdU-labeled cells we used the protocol for BrdU immunostaining of Bharali *et al*. [32]. Sections were incubated in 10% normal goat serum for 30 min, in rat anti-BrdU antibody (1: 50) (Oxford Biotechnology, Oxford, UK) overnight at 4°C (1∶200 in 10% normal goat serum) followed by goat-anti-rat-Alexa Fluor 488 secondary antibody (1∶150 in 10% normal goat serum). Non-immunized normal mouse or rat IgG was used as an immunohistochemical control.

To label the pool of granule cells proliferating in the external granule layer in the postnatal period, BrdU was intraperitoneally injected into P5 control and PDC-deficient female pups [33]. For the acute labeling study (after a single injection of BrdU at 1 mg/kg body weight), brains were processed 1 h after injection and used for immunodetection of Brdu and NeuN as described below. For the chronic labeling experiments, P5 control and PDC-deficient females were injected with BrdU (1 mg/kg body weight, 5 times 2 h apart), allowed to survive for 7 additional days (till P12) and their brains then processed as described above.

### BrdU Immunostaining

Consecutive coronal sections from brains of BrdU-injected animals were immunostained with an antibody against BrdU (rat monoclonal, Oxford Biotechnology, Oxford, UK) or double labeled with BrdU plus NeuN (NeuN mouse antibody, Chemicon, Temucula, CA) using the following protocol. After a rinse with PBS, slides were incubated in 2 N HCl at 37°C for 30 min followed by 10 min incubation with 0.1 M boric acid at room temperature. Normal goat serum was applied for 30 min after a wash with PBS and then sections were covered with a single or a mixture of two primary antibodies (BrdU, dilution 1∶250, NeuN 1∶200) overnight at 4°C. After a wash with PBS, the secondary antibodies were applied (goat-anti-rat Alexa Fluor 594, (Molecular Probes, Carlsbad, CA) for BrdU immunostained sections and a mixture of anti-BrdU antibody and a goat anti-mouse NeuN Alexa Flour 488 for double immunostained sections for 2 h. Slides were then incubated with 4′,6-diamidino-2-phenylindole dilactate (DAPI) (0.01 mg/ml, Molecular Probes, Carlsbad, CA), briefly washed with PBS and mounted with Immu-Mount.

### Locomotor Analysis

Locomotor activity was recorded by an infrared motion-sensor system (Kinder Scientific, San Diego, CA) fitted outside a standard plastic cage tub (42.5 cm×22.5 cm×19.25 cm). The tubs used for locomotor testing did not have shavings in them and clean tubs were used for each test session. Each locomotor chamber was located in a sound and light attenuating enclosure. Each enclosure was equipped with a wall mounted fan that provided masking noise and was illuminated by an 8 W light bulb (light output 450 lm). This arrangement provided a novel environment for locomotor testing. Two levels of infrared motion sensors were used (for recording horizontal and vertical movements. The sensors at the lower level consist of eight pairs along the long axis and five pairs along the short axis each spaced 5.5 cm apart and were used to determine the position of the animal. The sensors at the upper level were spaced 5.5 cm apart along the short axis and recorded vertical rearing movements. The activity-monitoring system monitors each of the beams at a frequency of 0.01 s to determine whether the beams were interrupted. The interruption of any beam not interrupted during the previous sample was interpreted as an activity score. Mice were placed into a novel locomotor test chamber for 2 h. Distance traveled and rearing were the dependent measures. Locomotor activity was tested only once. Statistical Analysis: Distance traveled and rearing were plotted as 12 10 min epochs and analyzed with a two factor mixed analysis of variance with group (Control, PDC-deficient) as the between subject factor and 10-min epoch as the within subject factor.

### Pre-pulse Inhibition (PPI) Analysis

Startle reactivity was measured using two chambers (SR-LAB; San Diego Instruments, San Diego, CA). Each chamber consisted of a clear non-restrictive Plexiglas cylinder resting on a platform inside a ventilated box. A high-frequency loudspeaker inside the chamber produced both a continuous background noise of 68 decibels (dB) and the 120-dB startle pulse. Vibrations of the Plexiglas cylinder caused by the whole-body startle response of the animal were transduced into analog signals by a piezoelectric unit attached to the platform. The PPI test session consisted of startle trials (pulse-alone), pre-pulse trials (pre-pulse+pulse), and no-stimulus trials (nostim). The pulse-alone trials consisted of a 40-ms 120-dB pulse of broad-band noise. PPI was measured by pre-pulse+pulse trials that consisted of a 20- ms noise pre-pulse, 100-ms delay, followed by a 40-ms 120-dB startle pulse (120-ms onset-to-onset interval). The acoustic pre-pulse intensities were 4, 8 and 16 dB above the 68-dB background noise (i.e. 72, 76 and 84 dB). The no stim trials consisted of background noise only. The test session began and ended with five presentations of the pulse-alone trial; in between, each acoustic or nostim trial type was presented 10 times in a pseudorandom order. There was an average of 15 s (range, 12–30 s) between trials. For the drug studies, the mice were placed into the startle chambers 30 min after each injection, and a 68-dB background noise level was presented for a 10-min acclimation period and continued throughout the test session. The level of PPI was calculated as a percentage score for each acoustic prepulse trial type: % PPI = 100 −{[(startle response for pre-pulse+pulse)/(startle response for pulse-alone)] × 100}. The magnitude of the acoustic startle response was calculated as the average response to all of the pulse-alone trials, excluding the first and last blocks of five pulse-alone trials presented. The mice were tested twice for PPI and the data were averaged across the two test session.

### Statistical Analyses

Results are expressed as means ± S.D. or S.E.M. of ‘n’ observations as indicated, and were statistically analyzed using SigmaPlot software (version 5.0, Point Richmond, CA) or with a two factor mixed analysis of variance with group for behavioral analysis. Unpaired Student’s t test was applied and a difference at 0.05% (P<0.05) was considered as significant.

## Results

### Breeding Outcome and Genotyping Analysis

**Figure 1:**
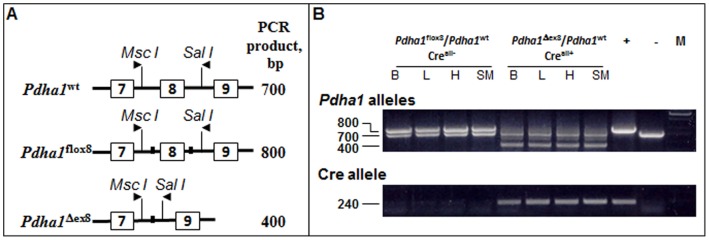
Genetic analysis of the *Pdha1* locus in control and PDC-deficient female progeny. (A) Depiction of wild-type (*Pdha1^wt^)*, floxed (*Pdha1^flox8^*), and null (*Pdha1* ^Δ*ex8*^) *Pdha1* alleles and the primers (arrows above the allele representations) used for genotypic analysis. (B) Genotypic analysis of the *Pdha1* locus in brain (B), liver (L), heart (H) and skeletal muscle (SM) tissues from a female offspring (genotye: *Pdha1* ^Δ*ex8*^ */Pdha1^wt^; Cre^all+^*) produced from mating a floxed homozygous female with a *Cre* transgenic male. Upper gel [genotypes: wild-type allele (700 bp), floxed allele (800 bp), and null allele (400 bp)]. Lower gel: [genotype: *Cre* transgene (240 bp)]. − (minus) Lane: DNA from a wild-type female (700 bp), and+(plus) Lane: floxed allele (800 bp; upper gel) from a floxed female and null allele (240 bp; lower gel) from a PDC-deficient female included as negative and positive controls. M: DNA marker.

The average litter size was significantly reduced by 33.8% when floxed females (genotype: *Pdha1^flox8^/Pdha1^flox8^*) were bred with transgenic males (genotype: *Pdha1^wt^/Y; Cre^all+^*) as compared to breeding with wild-type males that lacked the *Cre* transgene (Table 1). No *Cre*-positive male offspring were born to floxed females bred with the *Cre*-transgenic males (Table 1), consistent with their prenatal death reported in our earlier study [22]. *Cre*-positive heterozygous female offspring (PDC-deficient females), however, were born (n = 49) and used for metabolic and histological analyses (Table 1). PCR analysis of genomic DNA from brain, liver, heart and skeletal muscle of *Cre*-positive (PDC-deficient) females indicated the deletion of exon8 (400 bp; *Pdha1* ^Δ*ex8*^; from maternally-derived chromosome X) and wild-type *Pdha1* (700 bp; *Pdha1^wt^*; from paternally-derived chromosome X) alleles and the presence of *Cre* transgene (240 bp) in these tissues (Fig. 1A, B). The floxed allele (800 bp; *Pdha1^flox8^*) was not detected in these tissues (Fig. 1B). As expected, both the floxed and wild-type *Pdha1* alleles were detected in the same tissues from *Cre*-negative control females, while the deleted allele (400 bp, *Pdha1* ^Δ*ex8*^) and the *Cre* transgene (240 bp) were absent in the tissues from the control progeny.

**Table 1:** Sex distribution and genotype of progeny of floxed-females bred with wild-type and transgenic males.

Parameter	Breeding with wild-type males (n = 12)				Breeding with transgenic males(n = 11)			
Sex	Male		Female		Male		Female	
Genotype	*Cre^all^* ^−^	*Cre^all+^*	*Cre^all^* ^−^	*Cre^all+^*	*Cre^all^* ^−^	*Cre^all+^*	*Cre^all^* ^−^	*Cre^all+^*
# Pups	40	0	41	0	0	0	0	49
# Total	81				49			
Average litter	6.8±0.6^a^				4.50±0.5^b^			

a The results are means ± S.D. (n = 11–12).

b P<0.05.

### Body Weights and Plasma Substrate Levels

Body weights of PDC-deficient females of all ages were analyzed (E18, P0.5, P15 and P35) and did not significantly differ compared to age-matched control females. The average body weights of the control and PDC-deficient females were: 1.5±0.1 g and 1.6±0.1 g (n = 5) at age P0.5, and 20.5±0.5 g and 18.6±0.8 g (n = 8–13) at age P35, respectively. Brain weights of the PDC-deficient females were significantly lower compared with that from control females for ages E18 and P35 [at age E18: 53.1±2.0 mg, and 71.1±7.4 mg (n = 5), respectively, P<0.05; and at age P35: 381±17 mg, and 489±19 mg (n = 13), respectively, P<0.05]. Plasma lactate levels (2.8±0.1 and 2.6±0.4 mM, respectively) differed between randomly-fed P35 PDC-deficient and control females.

### PDC Activity in Tissues

**Figure 2:**
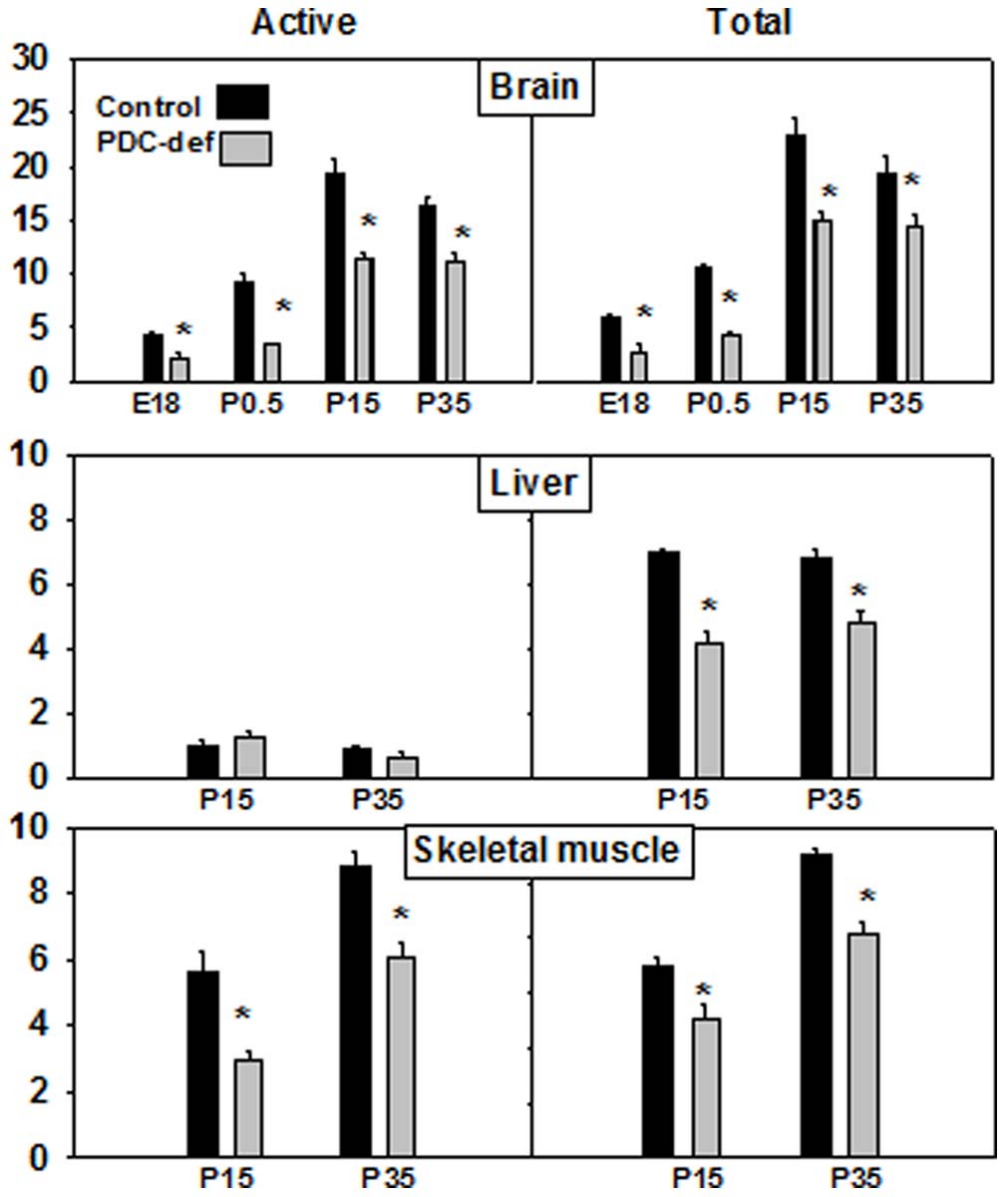
PDC activities in brain, liver and skeletal muscle of control and PDC-deficient females. PDC activity was measured as described in the Methods section. PDC activity is expressed as munits/mg cellular protein. The results are means ± S.D. for 4–6 animals.

‘Active’ and ‘total’ PDC activities were assayed in brain homogenates from control and PDC-deficient females at ages E18, P0.5, P15 and P35 (Fig. 2). In brain homogenates from PDC-deficient females at E18, P0.5, P15 and P35, ‘total’ PDC activity was significantly reduced by about 25–50% (P<0.05) compared to age-matched control females (Fig. 2). We also measured ‘active’ PDC activity to investigate if cells with reduced levels of ‘total’ PDC in the brain compensated by increasing their percent of ‘active’ non-phosphorylated PDC. In all age groups analyzed ‘active’ PDC was also significantly reduced in brain homogenates of PDC-deficient females compared to age-matched control females by about 25–50% (P<0.05) (Fig. 2).

Assays of ‘total’ PDC activity in liver homogenates at P15 and P35 revealed a decrease (∼25%, P<0.05) in PDC-deficient females compared to age-matched control females. No significant changes of ‘active’ non-phosphorylated form of PDC were observed in livers from P15 and P35 PDC-deficient females, possibly due to very low levels of ‘active’ PDC activity in their livers (Fig. 2). In homogenates of skeletal muscles analyzed at P15 and P35, both the ‘total’ and ‘active’ PDC activities were reduced (∼25–40%, P<0.05) in PDC-deficient females compared to age-matched control females (Fig. 2).

### Glucose Oxidation and Lipogenesis

To investigate the metabolic effects of reduced PDC activity in brains of PDC-deficient females at P15 and P35, we incubated brain slices with [U-^14^C]glucose or [1, 2-^14^C]acetate to assess oxidation of the substrates and their incorporation into fatty acids (Table 2). When [U-^14^C]glucose was used as a substrate, both oxidation and incorporation into fatty acids were significantly decreased in the brain slices from P15 PDC-deficient females compared to age-matched control females. As expected, metabolism of [1, 2-^14^C]acetate was similar for brain slices from both groups of P15 females. At P35, glucose oxidation in the brain slices was also significantly reduced in PDC-deficient females compared to age-matched control females. No changes in the incorporation of [U-^14^C]glucose-carbon into fatty acids was observed at this age group, possibly due to the lower rates of cerebral lipid synthesis in the post-weaning period [6]. Similar to P15 females, oxidation of [1, 2-^14^C]acetate and its incorporation in newly synthesized fatty acids did not differ significantly between the two groups of P35 females.

**Table 2:** Glucose and acetate oxidation to CO_2_ and glucose-carbon and acetate-carbon incorporation into fatty acids by brain slices from of P15 and P35 control and PDC-deficient females.

Age	Substrate	Parameter	Control	PDC-deficient
P15	[U-^14^C]glucose	CO_2_ formation	627±18^a^	535±10^b^
		Lipogenesis	54±1	32±3^b^
P15	[1,2-^14^C]acetate	CO_2_ oxidation	531±14	529±10
		Lipogenesis	74±8	64±4
P35	[U-^14^C]glucose	CO_2_ formation	886±39	569±30^b^
		Lipogenesis	14±1	13±1
P35	[1,2-^14^C]acetate	CO_2_ oxidation	617±18	585±20
		Lipogenesis	30±3	27±3

a Data are means (nmol of labeled substrate converted/g tissue/h) ± S.D. (n = 5–6).

b P<0.05.

### Analysis of PDH^+^ and PDH^−^ Cells

**Figure 3:**
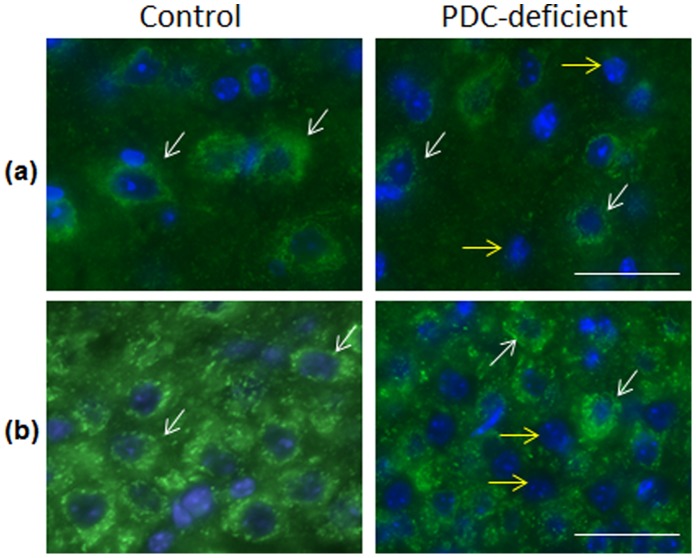
Immunodetection of PDHα. PDH (green) was detected using mouse monoclonal antibody (MitoSciences) and nuclear staining with DAPI (blue) in coronal sections of cerebral cortex (A) and cerebellar cortex (B) from P35 control female (left column) and PDC-deficient female (right column). Two types of cells are observed in composite to the brain structure of PDC-deficient females, (i) PDH^+^ cells with strong staining for PDHα (green) (examples: two cells are identified by white arrows) and (ii) PDH^−^cells without PDH staining (seen by the presence of their nucleus, stained with DAPI) (blue) (two cells are identified by yellow arrows. Bar is 100 µm.

Incubation of brain sections with a monoclonal antibody against PDHα resulted in specific labeling of cell cytoplasm but not nuclei (Fig. 3). Cells that were PDHα positive are referred to as PDH^+^ cells. In P35 control female brains all cells in all analyzed brain regions were PDH^+^. In P35 PDC-deficient females a significant number of brain cells in sections of cerebral neocortex (Fig. 3a upper panel, right column) and cerebellar cortex (Fig. 3b lower panel, right column) were not labeled with PDHα antibody. These cells were distinguished by the presence of their DAPI labeled nuclei and are referred to as PDH^−^ cells. A semiquantitative approach was used to assess PDH^+^ and PDH^−^ cells in P35 control and PDC-deficient females. As expected, no PDH^−^ cells were observed in control brains, while a significant number of PDH^−^ cells were found in PDH-deficient female brains (ranging from 17.4 to 65.3%, depending on the brain region) (Table 3). PDH^−^ cells were abundant in striatum, cortex and hippocampus while in cerebellum they were seen less often (Table 3). The proportion of PDH^−^ cells in the total population of cells varied among the brain regions and was highest in the striatum (65.3%) and lowest in the hippocampus (32.4%) (Table 3).

**Table 3:** Number of cells with PDH^+^ and PDH^−^ cells in brains of P35 control and PDC-deficient females.

Region	Control			PDC-deficient			
	PDH^+^	PDH^−^	Both cell types	PDH^+^	PDH^−^	Both cell types	%^c^
Cortex	7.5±0.2^a^	0	7.5±0.2	3.2±0.3^b^	3.0±0.3^b^	6.1±0.4^b^	49.2
Hippocampus	12.2±0.6	0	12.2±0.6	7.7±0.7^b^	3.6±0.4^b^	11.1±2.2^b^	32.4
Hypothalamus	10.2±0.6	0	10.2±0.6	6.0±0.4^b^	4.4±0.4^b^	10.3±0.4	42.7
Striatum	7.3±0.3	0	7.3±0.3	2.6±0.3^b^	4.9±0.4^b^	7.5±0.4	65.3
Cerebellum	4.0±0.2	0	4.0±0.2	3.0±0.3^b^	1.5±0.2^b^	4.5±0.2	33.3

a Data are means ± S.D.

b Significant difference (P<0.05) between the two groups of animals for a given type of cells examined.

c PDH^−^ as percent of total cells.

### Analysis of Brain Structures

**Figure 4:**
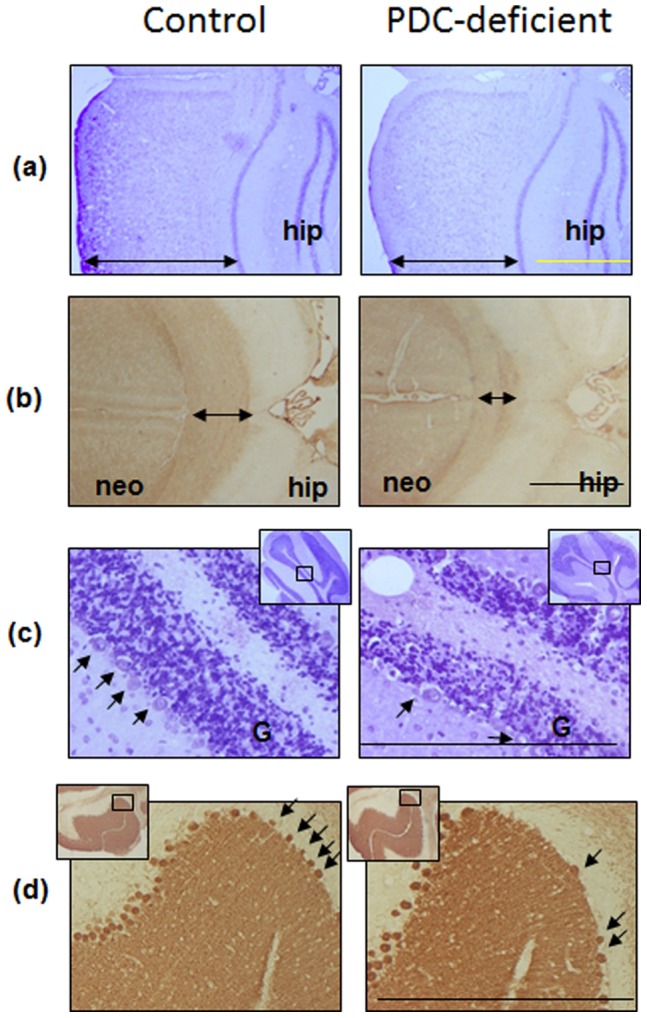
Detection of brain structures in coronal sections of control female and PDC-deficient female. (A) Cerebral neocortex (Nissl staining with Cresyl violet). Thickness of the neocortex (double arrow) is reduced in PDC-deficient female compared to control female. Fragment of the hippocampal formation (hip) is included for the orientation. (B) Corpus callossum (double arrow), Myelin Basic Protein immunostaining was thinner in PDC-deficient female compared to control female. Fragments of the neocortex (neo) and the hippocampal formation (hip) are included for the orientation. (C) Cerebellar cortex (Nissl staining with Cresyl violet) shows fewer Purkinje cells (arrows) and a thinner granule cell layer (G) in PDC-deficient female brain compared to control brain. Insets show lower magnification view on cerebellum and a small rectangle shows the area enlarged for the picture shown. (D) Cerebellar cortex (Calbindin D-28K immunostaining, monoclonal antibody) of PDC-deficient female illustrates fewer Purkinje neurons (arrows) compared with control female. Insets show lower magnification view on cerebellum and a small rectangle shows the area enlarged for the picture. Bar is 150 µm.

Histological analysis was performed using 20 µm frozen brain sections stained by (i) Nissl staining to reveal nuclei of neuronal and glial cells, (ii) anti-myelin basic protein to reveal myelinated structures, and (iii) anti-calbindin antibody to distinguish Purkinje neurons and their processes. Numerous changes in structures were found in white and grey matters of PDC-deficient females in comparison with control females. In sections stained with Cresyl violet the thickness of the neocortex was decreased in PDC-deficient females (Fig. 4a right). Occasionally, sites with irregular cytoarchitecture were observed. In these sites cells in PDC-deficient females were clustered in groups either radially or forming a thick horizontal layer and typical six-layered structure of the neocortex was not observed as in control females. The hippocampal formation of PDC-deficient females did not exhibit significant changes in structure (Fig. 4a). Incubation of brain sections with antibody against myelin basic protein resulted in specific labeling of white matter structures (Fig. 4b). In P35 PDC-deficient female brain, the majority of white matter structures were smaller in size than in age-matched control females. Among affected structures were the corpus callosum (Fig. 4b), anterior commissure (pars anterior), lateral olfactory tract, fibers of striatum and reticular thalamic nucleus, cerebellar fissures, and the pyramids (data not shown). The granule cell layer of cerebellar cortex (Nissl stained) was reduced in thickness and the density of granular neurons was decreased as well in PDC-deficient females (Fig. 4c, right). The density of Purkinje neurons was decreased (Fig. 4d).

### Quantification of Purkinje Neurons

**Figure 5:**
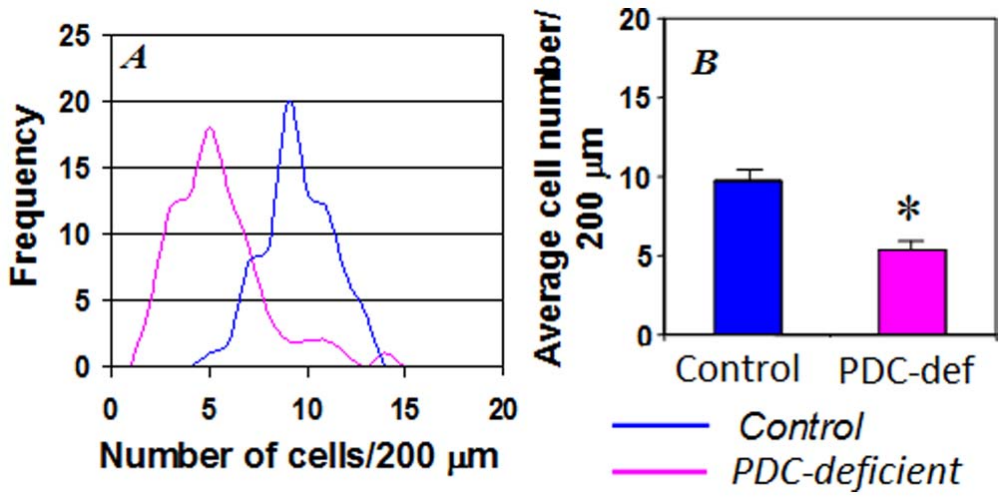
Quantitation of number of the Purkinje cell nuclei in P35 control and PDC-deficient females. (A) Gaussian histogram of the Purkinje cell nuclei counts. (B) Average counts of the Purkinje cell nuclei. Twenty sections from 2 brains from each group were analyzed. Sections were spaced at 350 mm. Area of the Purkinje cell layer was randomly chosen, its length was 200 µm. Data are means ± S.D., *represents P<0.05.

The number of Purkinje neurons in Nissl stained 20 µm thick cryosections spaced at 350 µm was quantified. Data were acquired from four different brains (2 control and 2 PDC-deficient females) in 80 random areas per cerebellum. A 200 µm length of the Purkinje neuron layer was chosen from different cerebellar folia and number of Purkinje neurons was quantified using Image Tool program, version 3.0. The mean number of Purkinje cells/200 µm for control brains was 9.75±0.23, and the number for PDC-deficient brains was 5.35±0.24 cells (P<0.05) (Fig. 5B). In addition, a frequency chart was constructed to characterize a range of low and high counts for Purkinje cells in control and PDC-deficient brains (Fig. 5A). Purkinje cells in a range 4–6 cells/200 µm were found in increased number in PDC-deficient brains whereas higher Purkinje cell counts ranging from 9–12 cells/200 µm were more often seen in increased number in control brains. These data show a reduction in Purkinje cell density in PDC-deficient brains.

### Differentiation of Purkinje Neurons

**Figure 6:**
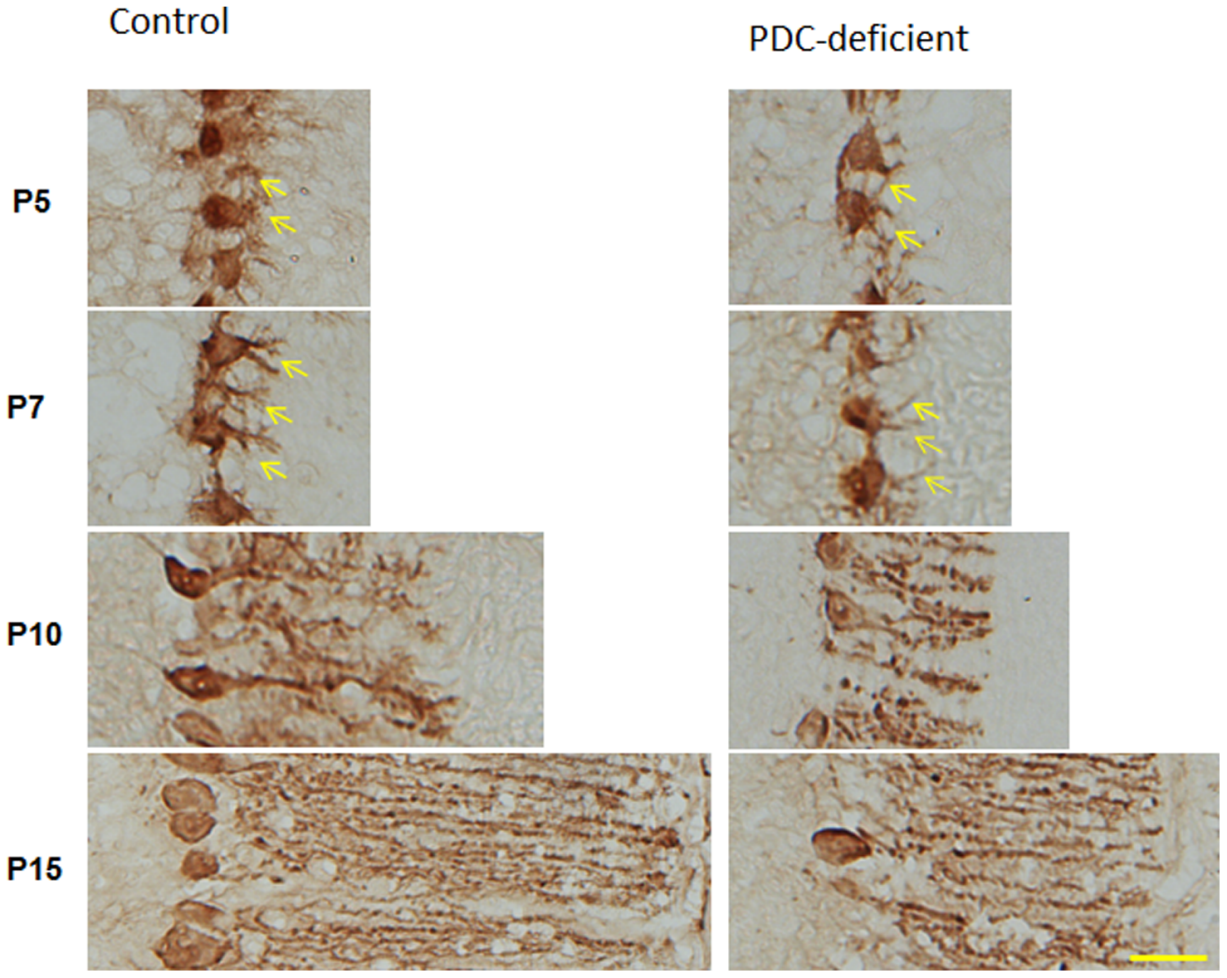
Photomicrograph of folia VIII (coronal section) from control and PDC-deficient females at different ages. Cryosections were immunostained using anti-calbindin D28K mouse monoclonal antibody (Sigma-Aldrich). Purkinje cells from PDC-deficient females at P5 showed fewer dendritic processes arising from cell body (yellow arrows in right column for P5) compared to age-matched control females. Purkinje cells from PDC-deficient females at P7 also showed fewer branches on dendrites (yellow arrows in right column for P7) compared to P7 control females. Purkinje cells from PDC-deficient females at P10 and P15 had shorter dendritic processes with fewer branches compared to controlled females. Bar is 10 µm.

Since Purkinje neurons differentiate postnatally from P5 to P17 [34], we examined the structure of the Purkinje neuron dendritic arbors at four different developmental ages (P5, P7, P10 and P15) (Fig. 6). Twenty µm thick frozen tissue sections, spaced 200 µm apart, were cut coronally and immunostained with anti-calbindin D-28K. Photomicrographs were obtained for the Purkinje neurons from cerebellar folia VIII. Compared to Purkinje neurons in brain sections from age-matched control females, Purkinje neurons from PDC-deficient females showed fewer dendritic processes at P5 (yellow arrows), fewer dendritic branches at P7 (yellow arrows), and shorter dendritic processes and fewer branches at P10 and P15. These findings clearly show impairment or delay in dendritic arbor development in Purkinje neurons from PDC-deficient females compared to control females (Fig. 6).

### Prenatal Cell Proliferation and Differentiation in Cerebellum

**Figure 7:**
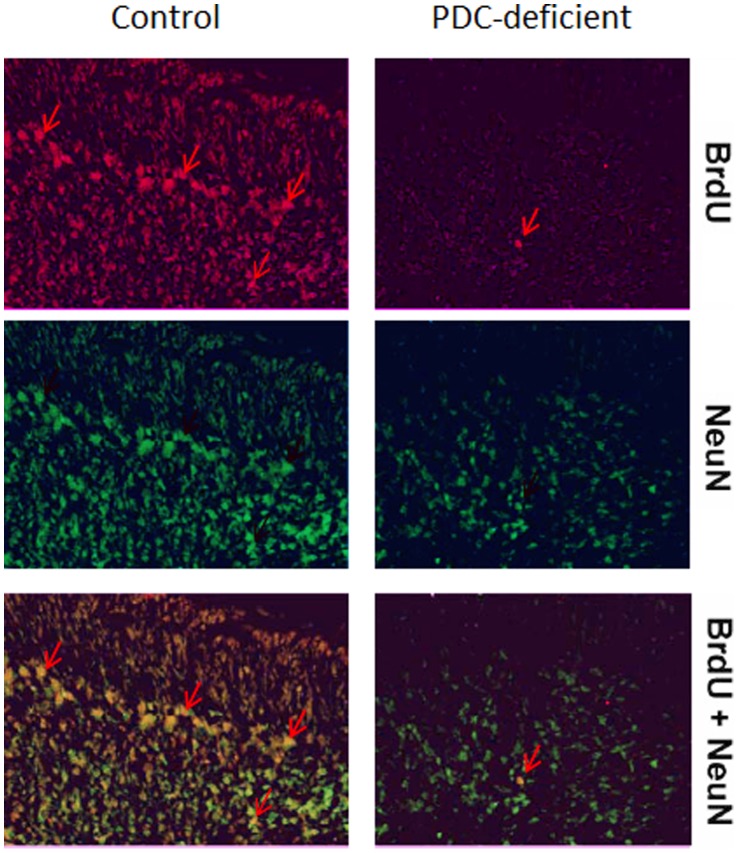
Immunostaining for BrdU and NeuN of the cerebella from P5 control and PDC-deficient females. BrdU was injected intraperitoneally (50 mg/kg body weight; 3 times 2 h apart), into 14-day pregnant dams. They were allowed to deliver pups naturally and nurse them. On P5 female progeny brain slices were immunolabeled with BrdU (red, upper panel) using rat anti-BrdU antibody (Oxford Biotechnology) and NeuN (green, middle panel) using mouse monoclonal antibody (Chemicon). Granule cell layer is marked by arrows. Bar is 50 µm.

To analyze differentiation of BrdU-labeled cells into neurons (NeuN^+^), multiple BrdU injections were performed in 14-day pregnant dams (50 mg/kg b.w.; 3 times 2 h apart, and dams were allowed to deliver and nurse their progeny. On P5 female progeny brains were processed and brain sections were co-immunostained for BrdU (Fig. 7, upper panel) and NeuU (middle panel). The precursors of neurons are known to be normally generated at E14 and then gradually differentiate to mature neurons. In P5 PDC-deficient cerebella, fewer BrdU^+^ granule neurons were observed compared to controls. Also, NeuN^+^ cells were also less abundant in the cerebella from PDC-deficient females (Fig. 7, middle panel). As expected, in control females more BrdU^+^/NeuN^+^ double-labeled cells (lower panel) were found indicating that the cells generated at E14 did differentiate into mature granule neurons. This finding indicated that the genesis of the neurons was impaired in in PDC-deficient cerebella.

### Postnatal Cell Proliferation and Differentiation in Cerebellum

**Figure 8:**
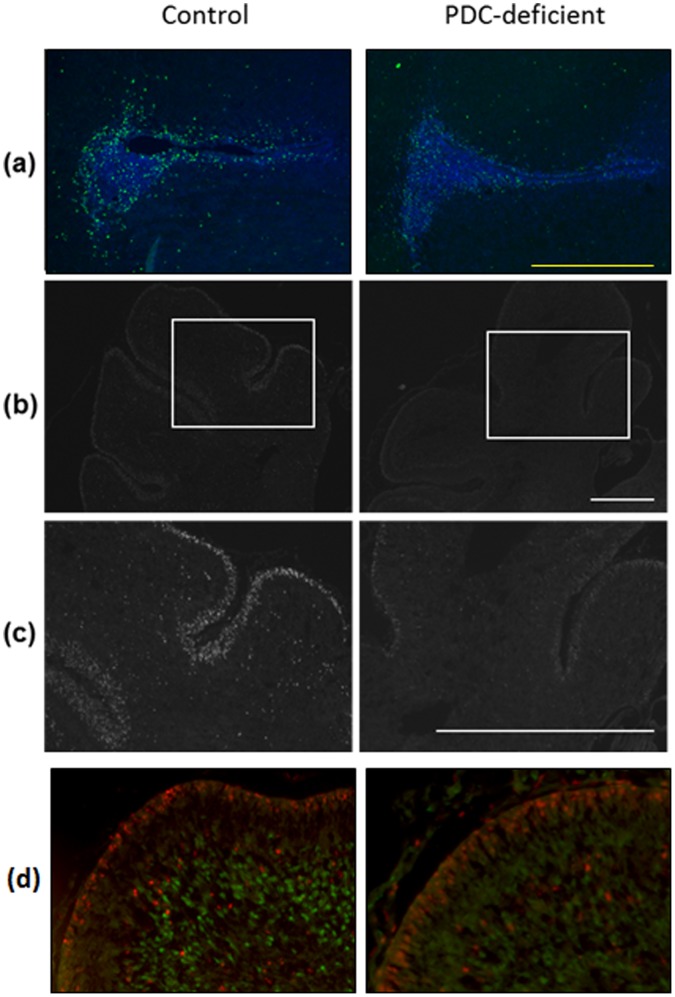
BrdU-immunostaining of coronal sections of the brain from P5 control and PDC-deficient females. After a single injection of BrdU (1 mg/kg body weight) brains were discected 1 h later and coronal sections were immunostained for BrdU and/or NeuN. (A) Lateral ventricle, (B) Cerebellar cortex, and (C) Magnification of a small block depicted in (B). Bar in a, b, and c is 100 µm. In a separate experiment (D), immunostaining was performed for BrdU (red) and counterstaining with NeuN (green) of a section of cerebellar cortex. Reduction in immunostaining for both the markers is seen in sections from PDC-deficient females compared to control females. As expected for an acute study, no co-localization is seen for BrdU^+^ cells and NeuN^+^ cells in both the groups.

To mark a pool of newly generated cells, we injected BrdU (1 mg/kg b.w., i.p.) [33] into P5 control and PDC-deficient female progeny. In the acute experiment, BrdU was injected once and pup brains were processed for the analysis after 1 h. Because BrdU incorporates into DNA of dividing cells, BrdU-positive cells (BrdU^+^) are thought to represent newborn cells. Many strongly BrdU^+^-labeled cells in lateral ventricle (Fig. 8a) and cerebellar cortex (Fig. 8b) were observed in acutely BrdU-injected control female brains. Weakly stained cells were also detected in these areas (Fig. 8 a, b). This latter group of cells might not have undergone the entire cycle of DNA synthesis within the short labeling period. In acutely BrdU-injected P5 PDC-deficient female brains, there was a lower density of strongly stained cells indicating a reduction in the number of dividing cerebellar cells (Fig. 8). In the lateral ventricle fewer brightly immunostained cells are seen in the subventricular zone of PDC-deficient female in comparison to control female (Fig. 8a). In the cerebellar cortex, there is a prominent decrease in the density of BrdU^+^ cells in the external granular layer in PDC-deficient females as well (Fig. 8b, c). PDC-deficient female brains were less dense in the cells stained intensely with BrdU in both germinal zones in comparison to control brains. In another experiment, we co-immunostained cerebellar sections with anti-BrdU antibody and anti-NeuN antibody (a marker of mature neurons). Immunostaining for both BrdU and NeuN were higher in the sections from P5 control females compared to age-matched PDC-deficient females (Fig. 8d). As expected, in this acute study no co-localization of BrdU and NeuN was observed in brain sections of control and PDC-deficient females (Fig. 8d).

**Figure 9:**
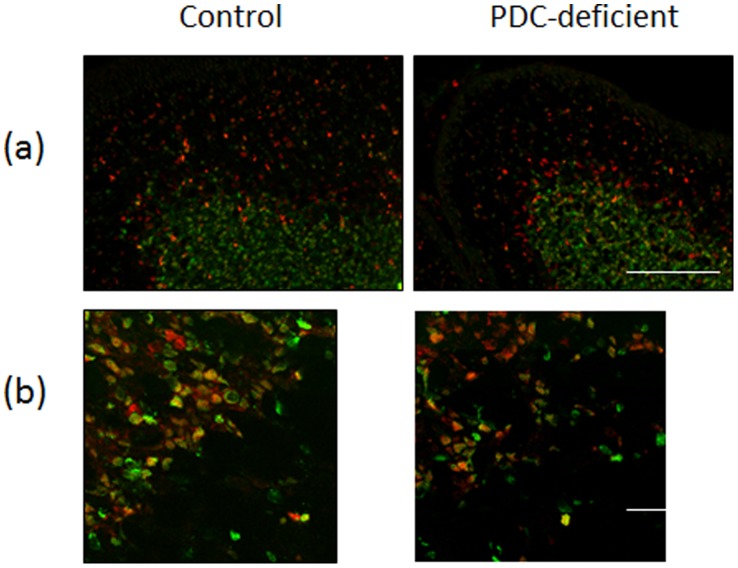
Immunostaining of coronal sections of cerebellar cortex from control females and PDC-deficient females. Pups were injected chronically with BrdU (1 mg/kg body weight, 5 times 2 h apart) on P5 and killed on P12. (A) This section shows immunostaing for BrdU (red) and NeuN (green). Images of the stained sections in (B) were taken with Nikon Optiphot BioRad 1024 Confocal Microscope (sequential mode). Fewer cells with co-localizing BrdU^+^ and NeuN^+^ (in yellow) are seen in PDC-deficient females, suggesting less differentiation of neurons in PDC-deficient females. Bar is 50 µm.

To assess proliferation over a longer period and to evaluate differentiation of the newly generated cells into mature neurons, one group of animals was subjected to multiple BrdU injections (5 injections of 1 mg/kg body weight every 2 h) on P5. In this experiment, pups were left to survive for 7 days after the last injection. In tissues with a higher rate of cell division a dilution of BrdU-labeled DNA results and hence the weakly BrdU^+^ cells likely represent cells that continued to proliferate after the last injection thus diluting the BrdU-labeled DNA [33]. On the other hand, in tissues with a lower rate of cell division, a higher level of incorporated BrdU in DNA would persist. A higher number of strongly BrdU^+^ cells were seen in PDC-deficient female cerebellar section as compared to a control female section. This suggests a lower proliferation rate in PDC-deficient female brain (Fig. 9a). When co-immunostained for NeuN, we observed a co-localization of BrdU and NeuN using a confocal microscope in the sections obtained 7 days after BrdU treatment (Fig. 9b). This co-localization was noticeably lower in PDC-deficient female brain compared to control female brain (Fig. 9b). This suggests a lower rate of differentiation of newborn cells into mature neurons in the brain of PDC-deficient females. Thus, the results of both the acute and chronic BrdU-labeling experiments suggest a lower proliferation rate in the brain of PDC-deficient females.

### Locomotor Analysis

**Figure 10:**
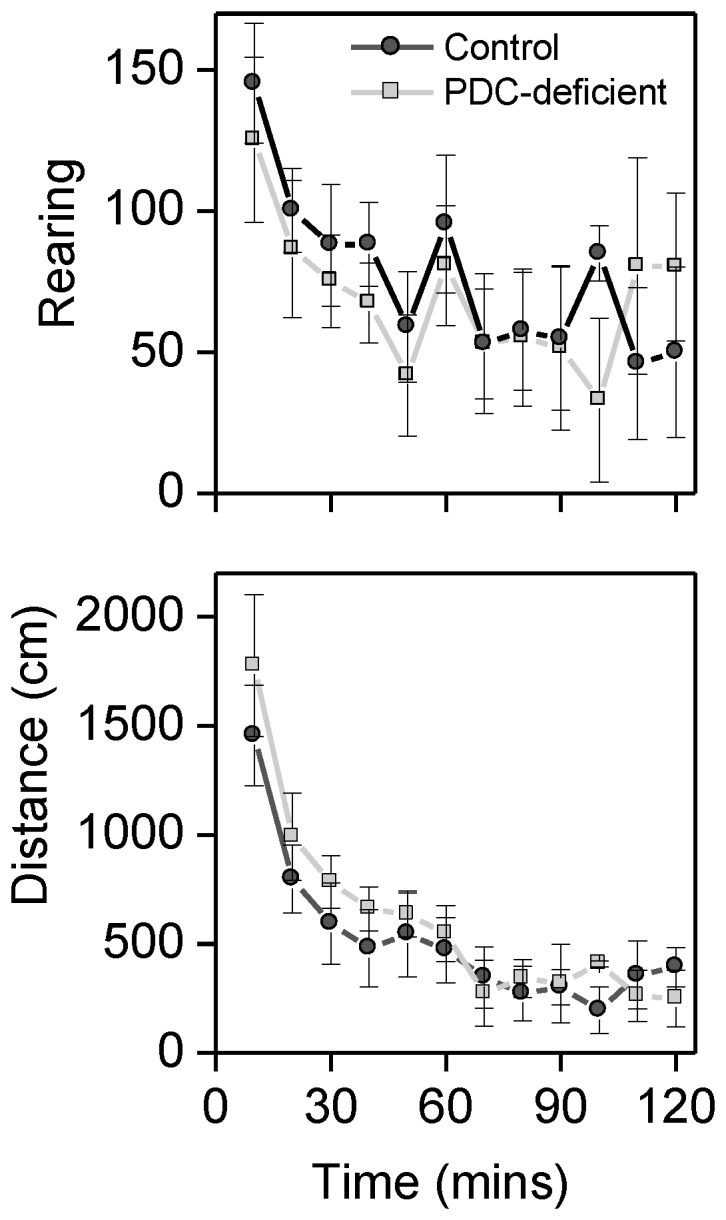
Each mouse was placed into a novel locomotor chamber and vertical movements (rearing) and horizontal movements (distance) recorded. PDC-deficient mice and control mice had equivalent activity levels and normal habituation to the novel environment. Data are reported as mean ± SEM (n = 4).

Distance traveled and rearing were plotted as 10 min epochs and analyzed with a two factor mixed analysis of variance with group (Control, PDC-deficient) as the between subject factor and 10-min epoch as the within subject factor. There was no effect of group on either distance traveled or rearing (Fig. 10). There was a significant effect of Epoch for both rearing (F11, 66 = 9.62, P<0.001) and distance traveled (F11, 66 = 30.29, P<0.001). These results indicate that both groups demonstrated a normal profile for locomotor activity in a novel environment. Activity levels were initially high and declined as the animals habituated to the novel environment.

### Startle Magnitude and Pre-pulse Inhibition

**Figure 11:**
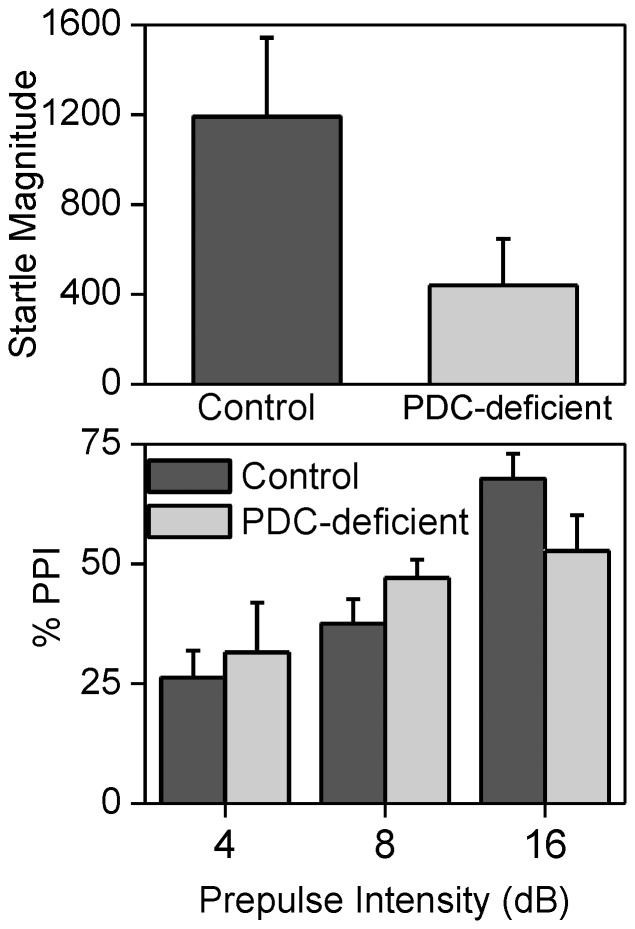
Plot a shows the acoustic startle response. Plot b shows pre-pulse inhibition of the acoustic startle response as a function the intensity of the pre-pulse stimulus. Pre-pulse intensity was measured in decibels (dB). PDC-deficient mice had markedly decreased startle responses and evidence of impaired pre-pulse inhibition of the acoustic startle response at the highest pre-pulse stimulus intensity. Error bars indicate standard error of the mean. Data are reported as mean ± SEM (n = 4).

Startle magnitude differences were analyzed using a one tailed between subject T-test. The PDC-deficient mice had a smaller startle magnitudes than the control group (t = 2.13, p<0.05, Fig. 11a). All four PDC-deficient animals had smaller startle magnitudes than the four control animals. Pre pulse inhibition was analyzed with a two factor mixed analysis of variance with group (Control, PDC-deficient) as the between subject factor and Pre-pulse amplitude (4, 8 and 16 dB above the 68-dB background noise) as the within subject factor. This analysis produced a significant effect of pre-pulse (F2, 12 = 32.15, P<0.001) and a significant pre-pulse by group interactions (F2, 12 = 5.63, P<0.05). However the source of the interaction is unclear because post-hoc t-tests found no significant between group differences at any pre-pulse levels. Examination of the Fig. 11b indicates that the most likely source of the significant interaction was that the PCA deficient animals had less pre-pulse inhibition at the 16dB pre-pulse level.

## Discussion

Although PDC deficiency results in malformation of brain structures and often severe neurological symptoms in PDC-deficient patients [8], [10], [11], the mechanisms responsible for aberrant brain development in affected patients remains largely uncharacterized. This is due primarily to the lack of availability of an animal model to investigate the effects of PDC deficiency on brain development and to test treatment options. A zebrafish model of PDC deficiency (resulting from deletion of dihydrolopiamide acetyltransferase activity) was developed to evaluate its effect on growth and efficacy of a ketogenic dietary treatment [21]. The findings of this study were interesting but were of limited applicability to human PDC deficiency due to a non-mammalian species origin of this model. The present study reports an additional mouse model of ‘systemic’ PDC deficiency presenting its central nervous system structural pathology that confirms what has already been reported for a mouse model with ‘cerebral’ PDC-deficiency [23], [35] and also extends the previous study by demonstrating abnormal cellular proliferation and migration especially in the cerebellum.

As shown in a previous study [22] and in the present study, no males with PDC deficiency were born under our breeding scheme indicating that null mutation in the *Pdha1* gene (localized on chromosome X) is lethal for male embryos. Indeed all reported male patients with PDC deficiency have a variable level of residual enzymatic activity ranging from 5–40% in cultured skin fibroblasts [9], [10]. As expected, heterozygous female mice with ‘systemic’ PDC deficiency were born under our breeding protocol. Surprisingly, compared to the previously reported model of ‘cerebral’ PDC deficiency, where about one half of all female embryos did not survive *in utero* [23], there is no indication of embryonic lethality for female embryos in the present study even though the deletion of exon 8 was initiated earlier on embryonic day 1 in the ‘systemic’ PDC deficiency model and despite having a similar degree of PDC deficiency in the brains from both the murine models. The reason for this unexpected difference between two models remains unexplained at present.

Although the deletion of *Pdha1* exon 8 was initiated in the ‘systemic’ murine model at an early embryonic stage [24], the percentages of reduction in PDC activity differed in different tissues. High variability in the reduction of PDC activity (25–50%) was also found in different regions of the brain of PDC-deficient females (Table 3) and also at different ages (Fig. 2). These findings suggest that mosaic expression of PDC deficiency took place due to random inactivation of chromosome X harboring *Pdha1* ^Δ*ex8*^. Immunolabeling of brain sections with anti-PDHα indeed showed the presence of two types of cells, PDH^+^ and PDH^−^ in the brains of PDC-deficient females (Fig. 3). Cells that expressed PDHα (PDH^+^ cells) were supposedly cells where X chromosome with the defective *Pdha1* ^Δ*ex8*^ allele was inactivated. Cells that did not express PDHα (PDH^−^ cells) were the cells where paternal *Pdha^wt^* allele was inactivated. Semi-quantification of PDH^+^ and PDH^−^ cells showed that different brain regions of the affected females had variable distribution of PDH^−^ cells. High but variable percentage of PDH^−^ cells (from 32.4% to 65.3%; Table 3) is consistent with the decrease in ‘total’ brain PDC activity ranging from 25 to 50% (in experimental females at different ages). This may account for regional and individual differences in inactivation of chromosome X containing *Pdha* ^Δ*ex8*^.

The reason why PDC deficiency has such devastating effects on the nervous system has not been determined, but is hypothesized to be due to the reliance of the brain on glucose as the principal energy source during both the prenatal and postnatal periods. Fetal rodent brain has low level of PDC activity and it increases rapidly during the suckling period reaching the adult level soon after weaning to support increased energetic and biosynthetic needs of the developing brain. [36]–[39]. Therefore, prenatal and immediate postnatal periods may represent the most vulnerable periods for PDC deficiency. The brain is comprised of several heterogeneous cell types with variable capacities for glucose metabolism. The glycolytic and oxidative capacities for glucose metabolism are differentially compartmentalized between astroglia and neurons which represent two major cell populations in the brain [40]–[42]. Astroglia metabolize glucose to lactate via the glycolytic pathway far in excess of its rate of oxidation in the mitochondria resulting in lactate release in the extracellular space. The PDC, in part, appears to be rate limiting step in the oxidation of pyruvate in astroglia. Neurons, on the other hand, readily oxidize both glucose and lactate to CO_2_ with the preference to oxidize external lactate (released by astroglia) over intracellular pyruvate and lactate generated from glucose via glycolysis [40]–[42]. PDC deficiency should affect both types of cells by reducing their overall capacity to generate energy from glucose and lactate. This prediction is supported by a significant reduction in glucose oxidation to CO_2_ by brain slices of PDC-deficient females on P15 and P35 (Table 2). Since glucose is the primary fuel for energy production in the brain, the reduction in glucose oxidation in PDC-deficient brains during development would cause deficits in energy production, possibly resulting in impaired cell proliferation and differentiation, as observed in this study. Furthermore, developing brain synthesizes largely its lipids *de novo* from acetyl-CoA derived from glucose metabolism via the PDC reaction. Our results on lipogenesis demonstrated that the biosynthesis of fatty acids from glucose-carbons by brains of P15 PDC-deficient mice was significantly reduced compared to control females (Table 2). This finding also supports the observed reduction in the brain weight of P35 PDC-deficient females. Interestingly, we did not find increased levels of blood lactate in PDC-deficient female mice. The reason for such an outcome is not clear. It is possible that the mild degree of PDC-deficiency (reduction in PDC-activity by about 25% only) in the mouse model as compared to much severe PDC deficiency in affected subjects (less than 50% and often 5–10% of control PDC activity) may account for such an outcome for the blood lactate levels. It should be noted that the reduction in PDC activity in all tissues from PDC-deficient female mice is due to mosaic expression of PDC activity (random inactivation of one of the two X chromosomes).

In the majority of reported cases of PDC deficiency, histo-pathological evaluation of the brain was not present. In selected cases of early neonatal morbidity, a common set of malformations has been identified. The most frequent observation is dilation of the cerebral ventricles, consistent with atrophy of cortex, and most pronounced laterally [8], [16], [17]. Underdevelopment of large white matter structures such as the corpus callosum, pons and pyramids have also been well described [11], [16]–[18], [43]. Atrophy or neuronal loss combined with gliosis has been often found in the cortex and less often in basal ganglia, thalamus, hypothalamus and cerebellum [16]–[18], [43]. A decrease in Purkinje neuron number was observed in cerebellar cortex [17]. Heterotopias have been found in all brain regions. Magnetic resonance imaging and post-mortem human studies have also shown profound decreases in the size of corpus callossum. Based on clinical findings and brain imaging data derived from 22 PDC-deficient subjects, Barnerias *et al.* [18] identified four different neurological presentations, namely malformations, acute brainstem dysfunction, congenital motor disorders, and relapsing ataxia. Analyses of brains of female mice with “systemic’ PDC deficiency showed that different brain regions had different degree of changes. White matter tissue showed a gross decrease in its size, as was observed in sections labeled with myelin basic protein (Fig. 4). Observed reduction in the neocortex thickness in P35 PDC-deficient females is consistent with the data from human patients where a gross reduction of cortical tissue and enlargement of lateral ventricles was often found [18]. Neuronal loss combined with gliosis was observed in PDC-deficient female mice in the present study. Furthermore, most of the observed structural abnormalities in the brain of systemic PDC-deficient mice (Fig. 4) are consistent with the reported pathologies in PDC-deficient patients. Our mice did not exhibit any basal ganglia lesions. This could be due to a milder degree of PDC-deficiency in our female mice. It should be noted that there are differences in the nature and degree of PDC-deficiency in the mouse model and affected patients. In our model with PDC-deficiency in heterozygous female mice only, each cell has either normal level of PDC activity or no activity at all (random inactivation of X chromosome). In PDC-deficient patients (both males and females) the nature of defect often reduces PDC activity (5–50%) but all cells have the same level of residual activity in male patients and variable level of residual activity in the cells of female patients. Furthermore, all our animals were 35-day-old at the time of pathological analysis whereas the reported PDC-deficient patients had a varying age from infancy to adolescent. These variations make direct comparison difficult to interpret.

Two main causes of structural defects of the central nervous system in PDC deficiency have been suggested: (i) developmental malformations and (ii) degenerative changes [7], [44]. Congenital developmental malformations have been described for all cases of PDC deficiency which were examined at autopsy, being remarkably less profound in males with early onset of disease [7]. However, destructive neurodegenerative lesions were more often observed in male patients with the early onset of the disease [44] suggesting that in males the primary cause may be reduced cell survival. Prenatal and early imaging in a few cases have suggested that the onset of these neurologic deficits occurs prenatally, however, the timing and pathologic progression are essentially uncharacterized in PDC-deficient patients [7], [8], [18], [45]. Our results also show that cell proliferation and differentiation are impaired in PDC-deficient fetuses (Fig. 7) (also see below).

To evaluate the cellular processes responsible for structural abnormalities, we analyzed BrdU incorporation into brain cells prenatally (E14) and postnatally (P5) using single injection to study cell proliferation and multiple injections with survival of the experimental animals to investigate cell migration and differentiation. The prenatal studies indicated impairment in proliferation of cells on E14 and their subsequent migration and differentiation into mature Purkinje neurons on P5 (Fig. 7). Studies with postnatally injected BrdU were used to address question whether reduction in cerebellar granule cell volume originated from changes in proliferation and migration processes. Using acute BrdU labeling protocol with P5 mice we found reduction in density of actively proliferating cells. Moreover, the results of chronic BrdU labeling experiment with P5 mice (and analysis of their brains at P12) showed increased density of BrdU^+^ cells in the layer intermediate between external and internal granule cell layers. These findings suggest reduced proliferation of granule cells and their delayed migration during the postnatal period. Taken together, the results show that both cell proliferation and differentiation of newly generated precursors into neurons are reduced in PDC-deficient female mice in both the prenatal and early postnatal periods.

There was no evidence of gross motor defects in PDC-deficient mice as measured by locomotor activity in a novel environment. PDC-deficient mice had markedly decrease acoustic startle reflexes and demonstrated evidence of impaired PPI. Clear evidence of deficits in PPI may have been masked by the impaired acoustic startle reflex of PDC-deficient mice and the small number of mice tested. The decreased startle response and evidence of impaired pre-pulse inhibition indicate impaired neurological function in PDC-deficient mice.

In summary, we have shown that systemic PDC deficiency in mice causes embryonic lethality for male only. Heterozygous PDC-deficient females show reductions in (i) brain weight, (ii) oxidation of glucose to CO_2_, (iii) *de novo* synthesis of fatty acids, and (iv) alterations in many structures of the brain. The results also show impairment in cell proliferation, migration and differentiation into neurons during the prenatal and postnatal periods, leading to structural abnormalities in the brain of PDC-deficient female mice.

## References

[1] Harris RA, Bowker-Kinley MM, Huang B, Wu P (2002) Regulation of the activity of the pyruvate dehydrogenase complex. Adv Enzyme Regul 42: 249–259. 12123719 10.1016/s0065-2571(01)00061-9

[2] Patel MS, Korotchkina LG (2003) The biochemistry of the pyruvate dehydrogenase complex. Biochemistry and Molecular Biology Education 31: 5–15.

[3] Maragos C, Hutchison WM, Hayasaka K, Brown GK, Dahl HH (1989) Structural organization of the gene for the E1 alpha subunit of the human pyruvate dehydrogenase complex. J Biol Chem 264: 12294–12298. 2745444

[4] Dahl HH, Brown RM, Hutchison WM, Maragos C, Brown GK (1990) A testis- specific form of the human pyruvate dehydrogenase E1 alpha subunit is coded for by an intronless gene on chromosome 4. Genomics 8: 225–232. 2249846 10.1016/0888-7543(90)90275-y

[5] Siess E, Wittmann J, Wieland O (1971) Interconversion and kinetic properties of pyruvate dehydrogenase from brain. Hoppe Seylers Z Physiol Chem 352: 447–452. 5550962 10.1515/bchm2.1971.352.1.447

[6] Patel MS, Tonkonow BL (1974) Development of lipogenesis in rat brain cortex: the differential incorporation of glucose and acetate into brain lipids in vitro. J Neurochem 23: 309–313. 4153857 10.1111/j.1471-4159.1974.tb04359.x

[7] Brown GK, Otero LJ, LeGris M, Brown RM (1994) Pyruvate dehydrogenase deficiency. J Med Genet 31: 875–879. 7853374 10.1136/jmg.31.11.875 PMC1016663

[8] Robinson BH (1995) Lactic Acidemia (Disorders of pyruvate carboxylase, pyruvate dehydrogenase). In: Scriver CR, Beaudet AL, Sly WS, Valle D, editors. Metabolic and Molecular Basis of Inherited Disease, Seventh Edition New York: McGraw-Hill. 1479–1499.

[9] Kerr DS, Wexler ID, Tripatara A, Patel MS (1996) Human Defects of the Pyruvate Dehydrogenase Complex. In: Patel MS, Roche TE, Harris RA, editors. Alpha-Keto Acid Dehydrogenase Complexes. Switzerland: Birkhauser. 249–269.

[10] Patel KP, O’Brien TW, Subramony SH, Shuster J, Stacpoole PW (2012) The spectrum of pyruvate dehydrogenase complex deficiency: clinical, biochemical and genetic features in 371 patients. Mol Genet Metab 105: 34–43. 22079328 10.1016/j.ymgme.2011.09.032 PMC3754811

[11] Debrosse SD, Okajima K, Zhang S, Nakouzi G, Schmotzer CL, et al (2012) Spectrum of neurological and survival outcomes in pyruvate dehydrogenase complex (PDC) deficiency: Lack of correlation with genotype. Mol Genet Metab 107: 394–402. 23021068 10.1016/j.ymgme.2012.09.001

[12] Imbard A, Boutron A, Vequaud C, Zater M, de Lonlay P, et al (2011) Molecular characterization of 82 patients with pyruvate dehydrogenase complex deficiency. Structural implications of novel amino acid substitutions in E1 protein. Mol Genet Metab 104: 507–516. 21914562 10.1016/j.ymgme.2011.08.008

[13] Lissens W, De Meirleir L, Seneca S, Liebaers I, Brown GK, et al (2000) Mutations in the X-linked pyruvate dehydrogenase (E1) alpha subunit gene (PDHA1) in patients with a pyruvate dehydrogenase complex deficiency. Hum Mutat 15: 209–219. 10679936 10.1002/(SICI)1098-1004(200003)15:3<209::AID-HUMU1>3.0.CO;2-K

[14] Quintana E, Gort L, Busquets C, Navarro-Sastre A, Lissens W, et al (2010) Mutational study in the PDHA1 gene of 40 patients suspected of pyruvate dehydrogenase complex deficiency. Clin Genet 77: 474–482. 20002461 10.1111/j.1399-0004.2009.01313.x

[15] Chow CW, Anderson RM, Kenny GC (1987) Neuropathology in cerebral lactic acidosis. Acta Neuropathol 74: 393–396. 3687391 10.1007/BF00687218

[16] Brown GK, Haan EA, Kirby DM, Scholem RD, Wraith JE, et al (1988) “Cerebral” lactic acidosis: defects in pyruvate metabolism with profound brain damage and minimal systemic acidosis. Eur J Pediatr 147: 10–14. 3123240 10.1007/BF00442603

[17] Michotte A, De Meirleir L, Lissens W, Denis R, Wayenberg JL, et al (1993) Neuropathological findings of a patient with pyruvate dehydrogenase E1 alpha deficiency presenting as a cerebral lactic acidosis. Acta Neuropathol 85: 674–678. 8337946 10.1007/BF00334680

[18] Barnerias C, Saudubray JM, Touati G, De Lonlay P, Dulac O, et al (2010) Pyruvate dehydrogenase complex deficiency: four neurological phenotypes with differing pathogenesis. Dev Med Child Neurol 52: e1–9. 20002125 10.1111/j.1469-8749.2009.03541.x

[19] Dahl HH, Hansen LL, Brown RM, Danks DM, Rogers JG, et al (1992) X-linked pyruvate dehydrogenase E1 alpha subunit deficiency in heterozygous females: variable manifestation of the same mutation. J Inherit Metab Dis 15: 835–847. 1293379 10.1007/BF01800219

[20] Brown RM, Dahl HH, Brown GK (1989) X-chromosome localization of the functional gene for the E1 alpha subunit of the human pyruvate dehydrogenase complex. Genomics 4: 174–181. 2737678 10.1016/0888-7543(89)90297-8

[21] Taylor MR, Hurley JB, Van Epps HA, Brockerhoff SE (2004) A zebrafish model for pyruvate dehydrogenase deficiency: rescue of neurological dysfunction and embryonic lethality using a ketogenic diet. Proc Natl Acad Sci U S A 101: 4584–4589. 15070761 10.1073/pnas.0307074101 PMC384790

[22] Johnson MT, Mahmood S, Hyatt SL, Yang HS, Soloway PD, et al (2001) Inactivation of the murine pyruvate dehydrogenase (Pdha1) gene and its effect on early embryonic development. Mol Genet Metab 74: 293–302. 11708858 10.1006/mgme.2001.3249

[23] Pliss L, Pentney RJ, Johnson MT, Patel MS (2004) Biochemical and structural brain alterations in female mice with cerebral pyruvate dehydrogenase deficiency. J Neurochem 91: 1082–1091. 15569252 10.1111/j.1471-4159.2004.02790.x

[24] Lakso M, Pichel JG, Gorman JR, Sauer B, Okamoto Y, et al (1996) Efficient in vivo manipulation of mouse genomic sequences at the zygote stage. Proc Natl Acad Sci U S A 93: 5860–5865. 8650183 10.1073/pnas.93.12.5860 PMC39152

[25] Kerr DS, Ho L, Berlin CM, Lanoue KF, Towfighi J, et al (1987) Systemic deficiency of the first component of the pyruvate dehydrogenase complex. Pediatr Res 22: 312–318. 3116495 10.1203/00006450-198709000-00015

[26] Noll F (1966) Methode zur quantitativen bestimmung von L-(+)-lactat- dehydrogenase und glutamat-pyruvat-transaminase. Biochem Z 346: 41–49.

[27] Lib MY, Brown RM, Brown GK, Marusich MF, Capaldi RA (2002) Detection of pyruvate dehydrogenase E1 alpha-subunit deficiencies in females by immunohistochemical demonstration of mosaicism in cultured fibroblasts. J Histochem Cytochem 50: 877–884. 12070266 10.1177/002215540205000701

[28] Paxinos G, Watson C (1986). The Rat Brain in Stereotaxic Coordinates. edition 2. New York: Academic Press.

[29] Takahashi T, Nowakowski RS, Caviness VS Jr (1996) Interkinetic and migratory behavior of a cohort of neocortical neurons arising in the early embryonic murine cerebral wall. J Neurosci 16: 5762–5776. 8795631 10.1523/JNEUROSCI.16-18-05762.1996 PMC6578981

[30] Takahashi T, Goto T, Miyama S, Nowakowski RS, Caviness VS Jr (1999) Sequence of neuron origin and neocortical laminar fate: relation to cell cycle of origin in the developing murine cerebral wall. J Neurosci 19: 10357–10371. 10575033 10.1523/JNEUROSCI.19-23-10357.1999 PMC6782435

[31] Hatta T, Moriyama K, Nakashima K, Taga T, Otani H (2002) The Role of gp130 in cerebral cortical development: in vivo functional analysis in a mouse exo utero system. J Neurosci 22: 5516–5524. 12097503 10.1523/JNEUROSCI.22-13-05516.2002 PMC6758239

[32] Bharali DJ, Klejbor I, Stachowiak EK, Dutta P, Roy I, et al (2005) Organically modified silica nanoparticles: a nonviral vector for in vivo gene delivery and expression in the brain. Proc Natl Acad Sci U S A 102: 11539–11544. 16051701 10.1073/pnas.0504926102 PMC1181239

[33] Ye P, Xing Y, Dai Z, D’Ercole AJ (1996) In vivo actions of insulin-like growth factor-I (IGF-I) on cerebellum development in transgenic mice: evidence that IGF-I increases proliferation of granule cell progenitors. Brain Res Dev Brain Res 95: 44–54. 8873975 10.1016/0165-3806(96)00492-0

[34] Sotelo C, Alvarado-Mallart RM, Frain M, Vernet M (1994) Molecular plasticity of adult Bergmann fibers is associated with radial migration of grafted Purkinje cells. J Neurosci 14: 124–133. 8283229 10.1523/JNEUROSCI.14-01-00124.1994 PMC6576836

[35] Pliss L, Mazurchuk R, Spernyak JA, Patel MS (2007) Brain MR imaging and proton MR spectroscopy in female mice with pyruvate dehydrogenase complex deficiency. Neurochem Res 32: 645–654. 17342409 10.1007/s11064-007-9295-z

[36] Hawkins RA, Williamson DH, Krebs HA (1971) Ketone-body utilization by adult and suckling rat brain in vivo. Biochem J 122: 13–18. 5124783 10.1042/bj1220013 PMC1176682

[37] Cremer JE, Heath DF (1974) The estimation of rates of utilization of glucose and ketone bodies in the brain of the suckling rat using compartmental analysis of isotopic data. Biochem J 142: 527–544. 4464840 10.1042/bj1420527 PMC1168317

[38] Wilbur DO, Patel MS (1974) Development of mitochondrial pyruvate metabolism in rat brain. J Neurochem 22: 709–715. 4407094 10.1111/j.1471-4159.1974.tb04284.x

[39] Takakubo F, Dahl HH (1994) Analysis of pyruvate dehydrogenase expression in embryonic mouse brain: localization and developmental regulation. Brain Res Dev Brain Res 77: 63–76. 7510589 10.1016/0165-3806(94)90214-3

[40] Itoh Y, Esaki T, Shimoji K, Cook M, Law MJ, et al (2003) Dichloroacetate effects on glucose and lactate oxidation by neurons and astroglia in vitro and on glucose utilization by brain in vivo. Proc Natl Acad Sci U S A 100: 4879–4884. 12668764 10.1073/pnas.0831078100 PMC153649

[41] Lam TK, Gutierrez-Juarez R, Pocai A, Rossetti L (2005) Regulation of blood glucose by hypothalamic pyruvate metabolism. Science 309: 943–947. 16081739 10.1126/science.1112085

[42] Schurr A (2006) Lactate: the ultimate cerebral oxidative energy substrate? J Cereb Blood Flow Metab 26: 142–152. 15973352 10.1038/sj.jcbfm.9600174

[43] Prick M, Gabreels F, Renier W, Trijbels F, Jaspar H, et al (1981) Pyruvate dehydrogenase deficiency restricted to brain. Neurology 31: 398–404. 6783978 10.1212/wnl.31.4.398

[44] Robinson BH, MacMillan H, Petrova-Benedict R, Sherwood WG (1987) Variable clinical presentation in patients with defective E1 component of pyruvate dehydrogenase complex. J Pediatr 111: 525–533. 3116190 10.1016/s0022-3476(87)80112-9

[45] Cross JH, Connelly A, Gadian DG, Kendall BE, Brown GK, et al (1994) Clinical diversity of pyruvate dehydrogenase deficiency. Pediatr Neurol 10: 276–283. 8068153 10.1016/0887-8994(94)90122-8

